# Sclerosing thymoma-like thymic amyloidoma with nephrotic syndrome: a case report

**DOI:** 10.1186/s13256-017-1370-8

**Published:** 2017-09-07

**Authors:** Yuto Kato, Miyuki Okuda, Koji Fukuda, Nobuya Tanaka, Akihiko Yoshizawa, Yoshinori Saika, Yoshisumi Haruna, Shouji Kitaguchi, Ryuji Nohara

**Affiliations:** 1 Federation of National Public Service Personnel Mutual Aid Association Hirakata Kohsai Hospital, 1-2-1, Fujisakahigashimachi, Hirakata, Osaka 573-0153 Japan; 2 Department of Diagnostic Pathology, Kyoto University Hospital, 54 Shougoin-Kawaharacho, Sakyo-ku, Kyoto 606-8507 Japan

**Keywords:** Sclerosing thymoma, Thymus, Amyloid, Nephrotic syndrome, Steroid

## Abstract

**Background:**

Primary localized amyloidosis presenting as an isolated mediastinal mass is extremely rare, especially in the thymus. Sclerosing thymoma is also an extremely rare anterior mediastinal tumor, pathologically characterized by extensive sclerotic lesions with hyalinization and calcification. Only 14 cases of sclerosing thymoma and five cases of thymic amyloidosis have been reported to date.

**Case presentation:**

A 78-year-old Japanese woman was diagnosed as having sclerosing thymoma (Masaoka stage IVa pericardial dissemination)-like thymic amyloidoma. She was diagnosed as having either lung cancer or mediastinal tumor with pericardial dissemination, and received palliative treatment. Three years later, she was readmitted with a complaint of general malaise. Since minimal change nephrotic syndrome was suspected based on the disease onset and selectivity index of urinary protein, steroid pulse therapy was started. Subsequently, because a marked reduction in tumor size was observed during maintenance treatment with prednisolone, a thoracoscopic needle biopsy was performed for a definitive diagnosis. According to the pathological findings and clinical investigations, a final diagnosis of sclerosing thymoma (Masaoka stage IVa pericardial dissemination)-like thymic amyloidoma was made.

**Conclusions:**

This is a case report of sclerosing thymoma-like thymic amyloidoma. Both sclerosing thymoma and thymic amyloidoma are extremely rare diseases: only 14 cases of sclerosing thymoma and five cases of thymic amyloidosis have been reported to date. In either diagnosis, our case is the first case in which marked reduction in tumor size was observed with steroid therapy. All reported cases of sclerosing thymomas underwent surgical resection, but steroid therapy to sclerosing thymoma has not been reported. It is still unknown whether steroid therapy is effective or not. The hyalinized components of sclerosing thymoma possibly contain amyloid deposits. The marked reduction in tumor size with steroid therapy may result in amyloid deposits. The association between sclerosing thymoma and thymic amyloidoma remains uncertain. Sclerosing thymoma should be stained with Congo red.

## Background

Primary localized amyloidosis presenting as an isolated mediastinal mass is extremely rare, especially in the thymus. Sclerosing thymoma is also an extremely rare anterior mediastinal tumor, pathologically characterized by extensive sclerotic lesions with hyalinization and calcification. Only 14 cases of sclerosing thymoma and five cases of thymic amyloidosis have been reported to date.

Amyloidosis can be classified as a systemic disease (80 to 90%) or as a localized disease (10 to 20%) [1]. Localized amyloidosis presenting as a mediastinal mass, especially in the thymus, is rare. Amyloidomas have been reported in multiple body sites, including: the respiratory, genitourinary, and gastrointestinal tracts; internal viscera (especially lung); skin; and breast. Amyloidoma or tumoral amyloidosis is a term that describes a mass in which amyloid deposits are present. An amyloid is defined by the biochemical nature of the protein in fibril deposits.

Sclerosing thymoma is an anterior mediastinal tumor, pathologically characterized by extensive sclerotic lesions with hyalinization and calcification. It is an extremely rare thymoma subtype, first reported in 1994. The clinical characteristics and causes remain unclear, and it is not well known. Tumor cell nests are not evident in the small specimens from needle biopsies. Since sclerosing thymoma consists of extensive hyalinization of fibrous tissue, it is difficult to obtain a definitive diagnosis. When hyalinized fibrous tissue is collected from a mass biopsy specimen in the anterior mediastinum, sclerosing thymoma and amyloidoma should be considered possibilities in the differential diagnosis. Special attention must be given to tumors found in the thymus gland that are composed primarily of fibrous tissue by, for example, preparing serial sections of the entire tumor, to ensure that minute thymomas are not overlooked. This case report describes a patient with sclerosing thymoma-like thymic amyloidoma that showed marked reduction in tumor size when steroids were administered for paraneoplastic minimal change nephrotic syndrome (MCNS).

## Case presentation

A 78-year-old Japanese woman presented with a chief complaint of general malaise. Her past medical history included hypertension, dyslipidemia, and rectal prolapse. She was on medication to treat hypertension and dyslipidemia, and their control was good. She did not have any history of autoimmune diseases, multiple myeloma, or dialysis treatment. She had never smoked tobacco. Her family history showed that her eldest son had colon cancer. Nobody in her family had a history of amyloidosis.

### Current medical history

In 2013, she first presented with dyspnea. Chest computed tomography (CT) showed a mass in her anterior mediastinum and cardiac tamponade. Following the removal of approximately 1200 ml of pericardial effusion by pericardial drainage, pericardial adhesion therapy was conducted. Class III (malignancy suspected) cells were identified from the pericardial effusion, leading to a diagnosis of either lung cancer or mediastinal tumor with pericardial dissemination. She received 1.0 mg of dexamethasone alone for palliative treatment because resection was not possible due to her age. While the size of the tumor increased gradually, she continued to receive out-patient care because she did not have any subjective symptoms. In June 2016, she was readmitted with a complaint of general malaise.

On admission, her height was 147.5 cm and weight 44.0 kg. She was lucid, with heart rate (HR) 88/minute, blood pressure (BP) 134/94 mmHg, peripheral oxygen saturation (SpO_2_) 98% (room air), and body temperature (BT) 37.1 °C. Her heart sounds were normal with no murmurs, decreased breath sounds in both lung fields, and no rales. Her abdomen was flat and soft, with no abdominal tenderness and normal bowel sounds. Clear pitting edema was observed in both forearms and legs.

**Fig. 1:**
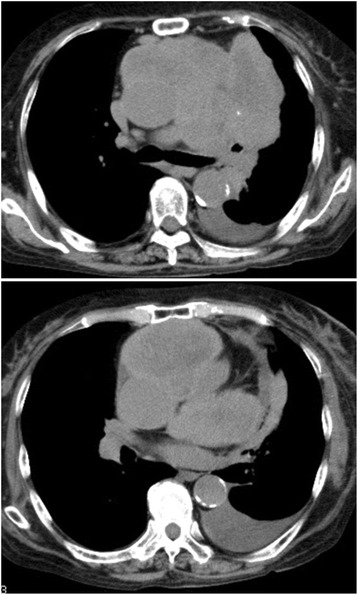
Computed tomography. Computed tomography revealed a 78×48 mm irregular-shaped tumor shadow extending from the *left* anterior mediastinum to the mid-mediastinum, in contact with the pericardium, along with copious pericardial fluid

Laboratory findings are shown in Table 1, and imaging findings are presented in Fig. 1.

**Table 1:** Blood and urine tests

**(Hematology)**		**(Antibodies)**		**(Tumor markers)**	
WBC	16800/mm^3^	Ach-R	<0.2 nmol/L	CEA	1.34 ng/mL
Neutro	6434/mm^3^ (38.3%)	IgG	567 mg/dL	SCC	1.1 ng/mL
Baso	67/mm^3^ (0.4%)	IgA	172 mg/dL	NSE	12.1 ng/mL
Eosino	16/mm3 (0.1%)	IgM	48 mg/dL	CYFRA	9.9 ng/mL
Lympho	9760/mm^3^ (58.1%)	IgD	<0.6 mg/dL	ProGRP	75.2 pg/mL
Mono	520/mm^3^ (3.1%)	IgG4	<3.0 mg/dL	s-IL2R	1038.9 U/L
RBC	493×10^4^/mm^3^	HTLV-1/CLEIA	(−)		
Hb	14.3 g/dL	EBV antiVCA-IgG	20 times	**(Urine analysis)**	
PLT	27.4×10^4^/mm^3^	EBV antiVCA-IgM	<10 times	pH	5.5
		EBV antiEA-IgG	10 times	Glucose	(−)
**(Biochemistry)**		EBV antiEBNA	<10 times	Protein	(4+)
TP	5.9 g/dL	HIV-Ag/Ab	0.06 S/CO	Blood	(+/−)
Alb	1.7 g/dL	HIV	(−)	RBC	1~4/HPF
T-Bil	0.42 mg/dL			WBC	30~49/HPF
AST(GOT)	18 IU/L	**(Immunoelectrophoretic study)**		Hyaline cylinder	20~29/LPF
ALT(GPT)	9 IU/L	(*Qualitative assay*)		Bence Jones protein	(−)
LDH	253 IU/L	Prealbumin	Normal	Protein/Cre ratio	16.46
ChE	315 IU/L	Albumin	Slightly low	Selectivity Index	0.017
ALP	223 IU/L	α1-Antitrypsin	Normal		
γ-GTP	13 IU/L	Haptoglobin	Normal		
CK	43 IU/L	α2-Macroglobulin	Normal		
T-Chol	431 mg/dL	β -Lipoprotein	Normal		
BUN	89.3 mg/dL	Transferrin	Normal		
Cre	1.13 mg/dL	Hemopexin	Normal		
Na	131 mEq/L	β-1C/β-1A globulin	Normal		
K	5.2 mEq/L	IgG	Slightly low		
Cl	97 mEq/L	IgA	Normal		
Ca	7.9 mg/dL	IgM	Normal		
IP	5.7 mg/dL				
TSH	1. 59 μIU/mL				
FT4	1.00 ng/dL				
CRP	0.26 mg/dL				

The patient had hypoalbuminemia. Minimal change nephrotic syndrome was suspected based on selectivity index. The anti-acetylcholine receptor antibody was negative. *Ach-R* acetylcholine receptor antibody, *Ag/Ab* antigen/antibody, Alb albumin, *ALP* alkaline phosphatase, *ALT(GPT)* alanine aminotransferase (glutamate-pyruvate transaminase), *AST(GOT)* aspartate aminotransferase(glutamic oxaloacetic transaminase), *BUN* blood urea nitrogen, *Ca* calcium, *CEA* carcinoembryonic antigen, *ChE* cholinesterase, *CK* creatine kinase, *CL* chlorine, *Cre* creatinine, *CRP* C-reactive protein, *CYFRA* cytokeratin fragment, *EA* early antigen, *EBNA* Epstein–Barr virus nuclear antigen, *EBV* Epstein–Barr virus, *FT4* free thyroxine, *γ-GTP* gamma-glutamyl transpeptidase, *Hb* hemoglobin, *HIV* Human Immunodeficiency Virus , *HTLV-1/CLEIA* human T-cell lymphotropic virus type 1/chemiluminescence enzyme-linked immunoassay, *IgG* immunoglobulin G , *IP* inositol monophosphate, *K* potassium, *LDH* lactate dehydrogenase, *Na* sodium, *NSE* neuron-specific enolase, *PLT* platelets, *Pro-GRP* pro-gastrin-releasing peptide, *RBC* red blood cell, *SCC* squamous cell carcinoma, *S/CO* sample-to-cutoff ratio, *s-IL2R* soluble interleukin-2 receptor, *T-Bil* total bilirubin, *T-Chol* total cholesterol, *TP* total protein, *TSH* thyroid-stimulating hormone, *VCA* viral capsid antigen, *WBC* white blood cell

### Clinical progress after admission

**Fig. 2:**
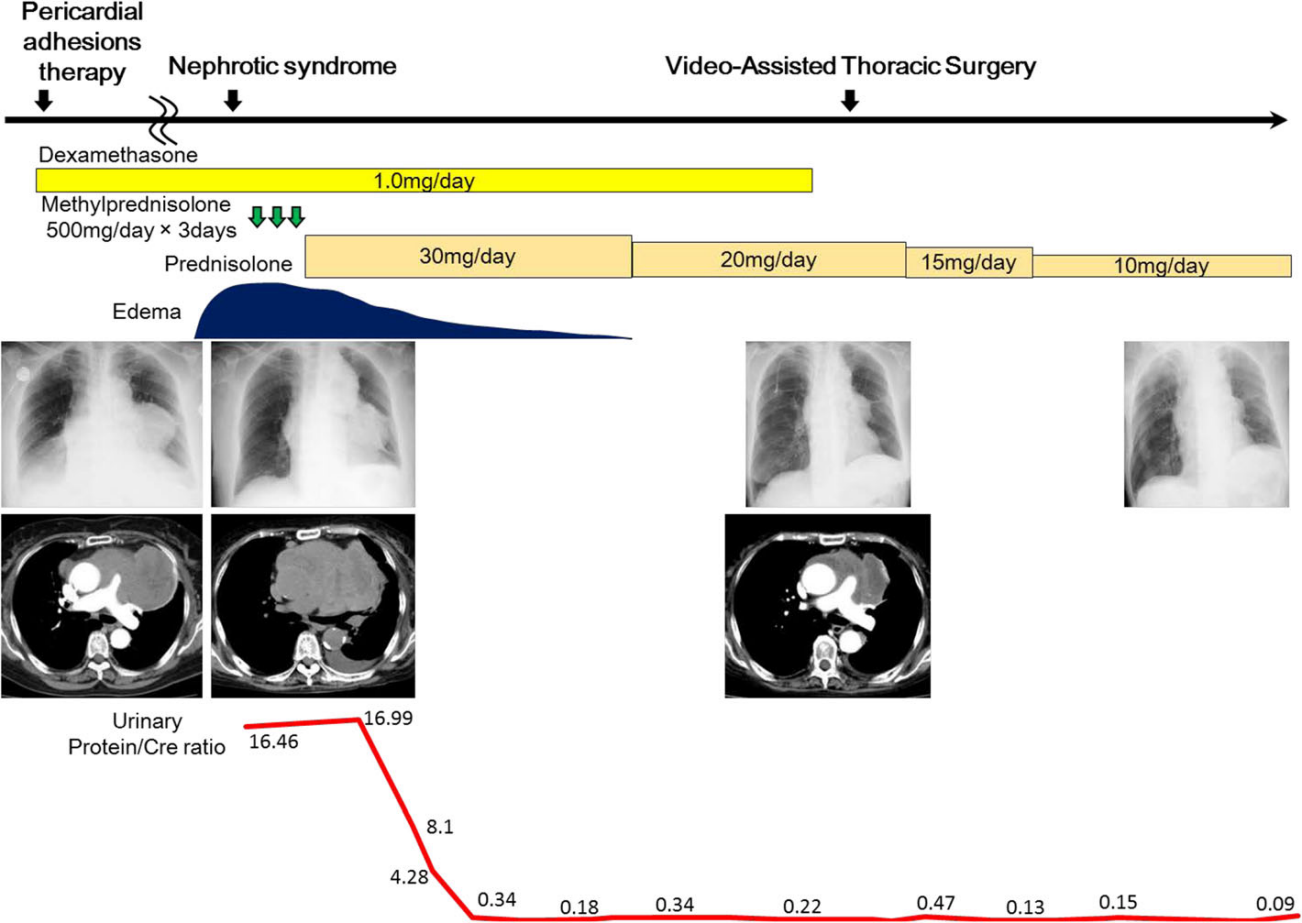
Clinical progress. After steroid pulse therapy, marked reduction in tumor size was observed

Blood and urine tests confirmed nephrotic syndrome. Since MCNS was suspected based on the disease onset and selectivity index of urinary protein, steroid pulse therapy (500 mg methylprednisolone/day × 3 days) was started. Edema of her legs reduced gradually and urinary protein excretion decreased. Subsequently, since a marked reduction in tumor size was observed and her general condition improved during maintenance treatment with 30 mg prednisolone, a thoracoscopic needle biopsy was performed in September 2016 for a definitive diagnosis. The pathological findings did not show malignancy, and prednisolone was tapered and maintained at 10 mg. Enlargement of the tumor and relapse of nephrotic syndrome has not been observed, and it remains under observation (Fig. 2).

### Radiological findings

**Fig. 3:**
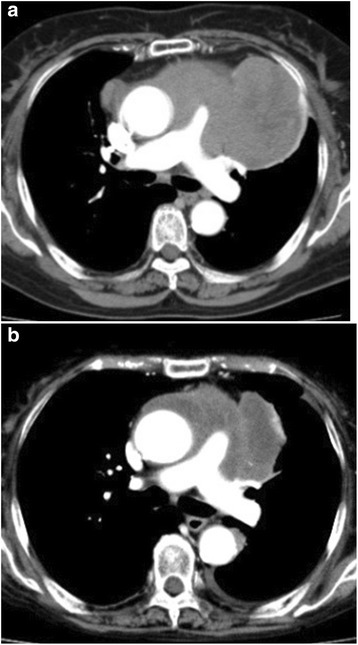
Enhanced computed tomography. **a** and **b** are both enhanced computed tomography images. The tumor was well enhanced when it was first found in 2013 (**a**). The tumor size reduced markedly, and the tumor changed into a poorly enhanced mass after steroid pulse therapy in 2016 (**b**)

A CT scan revealed a mass with nodular calcification in her left anterior mediastinum (Fig. 1). The tumor was well enhanced when it was first found in 2013 (Fig. 3a); the tumor was rich in epithelial cells and lymphocytes. However, the tumor changed into a poorly enhanced mass after steroid pulse therapy in 2016 (Fig. 3b). Decreasing enhancement of a mass might indicate that cells in the mass had disappeared and they were substituted by hyalinization after steroid therapy.

### Pathological findings

**Fig. 4:**
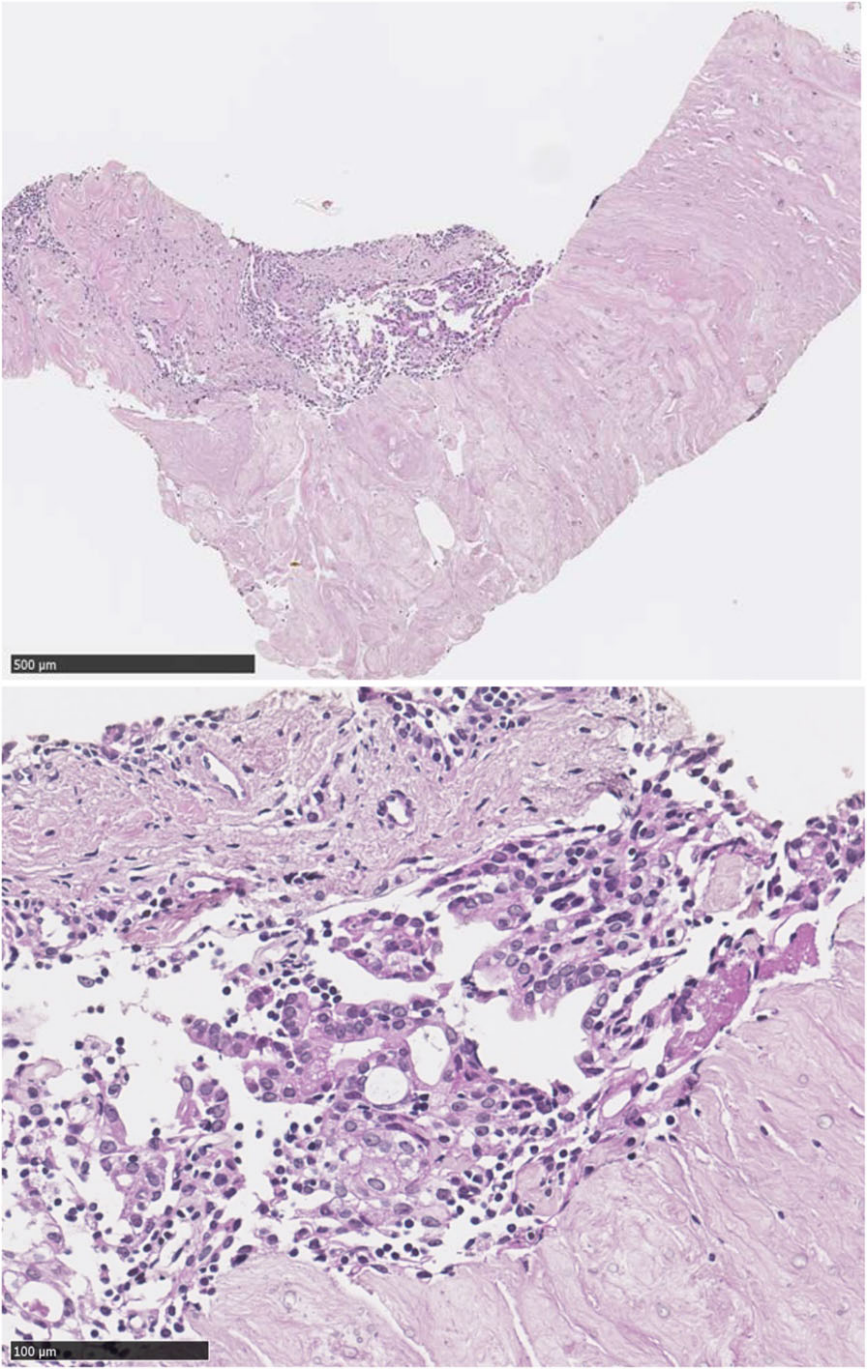
Biopsy specimens stained with hematoxylin and eosin stain. Biopsy specimens show dense hyalinized-collagenous tissue with focal epithelial cells intermingling with lymphocytes. The lymphocytes were positive for terminal deoxynucleotidyl transferase; thus, the epithelial cells were considered to be of thymic origin

**Fig. 5:**
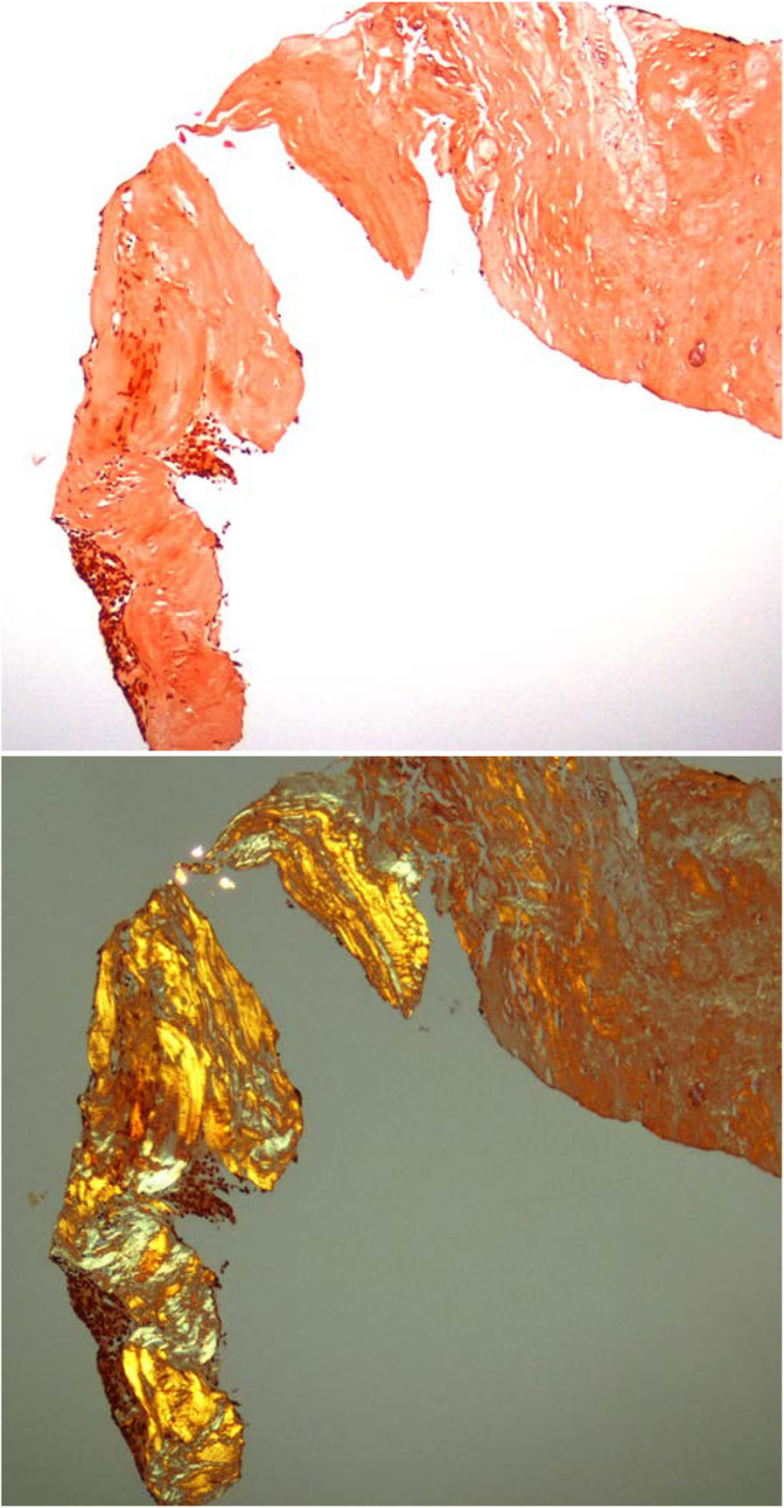
Biopsy specimens stained with Congo *red*. Thymic tissue is surrounded by congophilic amyloid. The hyalinized components of the tumor showed *apple-green* birefringence under polarized light after staining Congo *red*

A pathological examination showed mainly hyalinized components and, to a smaller extent, agglomeration of cellular components. A clear nucleolus and relatively abundant acidophilic cytoplasm as cellular components indicated the fusion of epithelioid cells. The epithelioid cell clusters were AE1/AE3-positive, thyroid transcription factor-1 (TTF-1)-negative, and calretinin-negative. The surrounding small lymphocytes were terminal deoxynucleotidyl transferase (TdT)-positive. Therefore, the expressed epithelial cells were considered to be thymus-derived cells. The expressed epithelial cells showed weak atypia, and there was no atypia of lymphocytes. Epstein–Barr virus *in situ* hybridization (EBV-ISH) was negative (Fig. 4). The hyalinized components of the tumor showed apple-green birefringence under polarized light after staining Congo red, which means the hyalinized components are amyloid deposits (Fig. 5). However, the epithelial cells in the specimens are TdT-positive thymus-derived cells, which cannot be explained one-dimensionally as amyloidoma. Immunohistochemical analysis was negative for kappa and lambda light chain, as well as amyloid A amyloidosis (AA amyloidosis). Transthyretin (TTR) amyloidosis deoxyribonucleic acid (DNA) sequencing was inconclusive for DNA sequence alteration in the coding region of the TTR gene. Combined with immunoelectrophoretic pattern, it indicated a low likelihood of primary amyloidosis.

Based on these findings, a final diagnosis of sclerosing thymoma (Masaoka stage IVa pericardial dissemination)-like thymic amyloidoma was made.

## Discussion

This case report presented a patient with sclerosing thymoma-like thymic amyloidoma that showed marked reduction in tumor size when steroids were administered for paraneoplastic nephrotic syndrome. Our patient was receiving palliative treatment because she could not undergo resection due to her age and pericardial dissemination.

The most common types of amyloidosis are light chain amyloidosis (AL amyloidosis) and AA amyloidosis. AL amyloidosis is associated with an underlying monoclonal plasma cell disorder. AA amyloidosis is associated with chronic inflammatory conditions, such as autoimmune diseases. In our case, our patient did not have any history of autoimmune diseases, multiple myeloma, or dialysis treatment. Nobody in her family had a history of amyloidosis. Immunohistochemical studies were negative both for kappa and lambda light chains and for AA amyloidosis. M protein was negative, and there was no evidence of systemic amyloidosis in clinical investigations.

Amyloidomas in the mediastinum are extremely rare, especially in the thymus. Only five cases of thymic amyloidosis have been reported to date [2–6] (Table 2). The average age of the five cases of thymic amyloidosis reported so far was 53 years (33 to 85 years). One of the cases was male and the other four were female. There may be no clinical symptoms in some cases. Tumor diameter was 6.3 cm on average, and total thymectomy was performed in all cases. Four cases were positive for AL amyloidosis or AA amyloidosis; however, there was a case (Case number 5) that was negative both for AL amyloidosis and AA amyloidosis. Two cases were complicated with myasthenia gravis, and they had steroid therapy before surgery. Our case showed a marked reduction in tumor size after steroid therapy, but information was not available on whether the reported two cases showed a reduction in tumor size after steroid therapy.

**Table 2:** Five cases of thymic amyloidosis and our case

Case number	Age (years)	Gender	Clinical symptom	Past history or underlying disease	Tumor size (cm)	Calcification/Ossification	Lymphoplasma cell infiltration	Amyloid type	Therapy
1	33	Female	(−)	RA	8.3	(+)/(+)	(−)	AA(+)	Surgical resection
2	55	Female	(−)	(−)	7	(+)/(+)	(+)	AL(+)	Surgical resection
3	46	Female	Ptosis, weakness in the neck, dyspnea	MG, myasthenic crisis	4	(+)/(+)	(+)	AL(+)	Immunoglobulin therapy, steroid therapy, surgical resection
4	85	Male	Diplopia	DM, HT, BPH, maxillary sinusitis, arthritis of the knee joint	4	(+)/NA	NA	AL(+)	Surgical resection
5	45	Female	Ptosis, diplopia	MG	8.4	(+)/NA	NA	AL(−) AA(−)	Steroid therapy and surgical resection
Our Case	78	Female	General malaise	HT	7.8	(+)/(−)	(−)	AL(−) AA(−)	Steroid therapy

Four cases were positive for light chain amyloidosis or amyloid A amyloidosis; however, there was a case (Case number 5) that was negative both for light chain amyloidosis and amyloid A amyloidosis. Two cases were complicated with myasthenia gravis, and they had steroid therapy before surgery. *AA* amyloid A amyloidosis, *AL* light chain amyloidosis, *BPH* benign prostate hypertrophy, *DM* diabetes mellitus, *HT* hypertension, *MG* myasthenia gravis, *NA* not available, *RA* rheumatoid arthritis

Sclerosing thymoma is a rare thymoma subtype, which was first reported in 1994 by Kuo [7]; 14 cases have been reported to date. The clinical characteristics and causes remain unclear, and even its existence is not well known. On histological examination, it is characterized by a lesion that has extensive hyalinization of fibrous tissue [8]. The differential diagnosis for an anterior mediastinal mass with extensive fibrosis includes: solitary fibrous tumor, Hodgkin’s lymphoma (nodular sclerosing type), mediastinal diffuse large cell lymphoma with sclerosis, and amyloidoma. The different type of fibrosis for each of the tumors becomes important for differential diagnosis. A solitary fibrous tumor is characterized by proliferation of keloid-like collagen fibers around cluster of differentiation (CD) 34-positive cells. Hodgkin’s lymphoma (nodular sclerosing type) is characterized by multinodular arrangements of birefringent collagen fibers around the lesions, and the presence of lacunar cells. Mediastinal diffuse large cell lymphoma has a characteristic histology known as compartmentalization, where each tumor cell is surrounded by hyalinized fibrous components. Thymoma itself may at times show strong fibrosis; however, strongly convoluted hyalinized fibrosis is a characteristic of sclerosing thymoma [9]. In the present case, sclerosing thymoma is one of the differential diagnoses because the tumor consisted of strongly convoluted hyalinized fibrosis and epithelial cells possibly derived from the thymus. The 14 cases of sclerosing thymoma reported so far had an average age of 49 years (23 to 73 years); our case was the eldest. There were seven males and seven females. There may be no clinical symptoms in some cases. Chest pain and dyspnea may occur, and patients with myasthenia gravis may have muscle weakness. Three cases were complicated with myasthenia gravis. Tumor diameter was 5.6 cm on average, and total thymectomy was performed in all cases (Table 3). Tumor cell nests are not evident in the small specimens from needle biopsies because sclerosing thymoma consists of extensive hyalinization of fibrous tissue [8]. It is difficult to obtain a definitive diagnosis. The present case was diagnosed by thoracoscopic needle biopsy. The intraoperative findings showed firm adhesion of the left S1+2 and the mediastinal side. However, as induration was felt at the same location, four needle biopsies were performed using 20-gauge single-use tissue biopsy needle. When hyalinized fibrous tissue is collected from a biopsy of an anterior mediastinal mass, sclerosing thymoma should be considered one of the possibilities in the differential diagnosis. Special attention must be given to tumors found in the thymus gland that are composed primarily of fibrous tissue by, for example, preparing serial sections of the entire tumor, to ensure that minute thymomas are not overlooked. Relating to needle biopsy, fine needle aspiration techniques usually suffice for carcinomatous lesions but a cutting needle biopsy should be performed whenever possible. It is important to obtain larger specimens for a more accurate histological diagnosis [10]. Using 18 to 20-gauge biopsy needle, the diagnostic sensitivity and specificity of CT-guided percutaneous cutting needle biopsy for thymic tumors were 93.3 and 100% [11]. Therefore, our diagnosis is considered to be quite accurate.

**Table 3:** Fourteen cases of sclerosing thymoma and our case

Case number	Age (years)	Gender	Clinical symptom	Myasthenia gravis	Tumor size (cm)	Biopsy	Follow-up
1	39	Female	Palpitation, dyspnea	(+)	3.0	Surgical resection	Well, 4 years
2	23	Female	Muscle weakness	(+)	2.5	Surgical resection	Well, 2 years
3	34	Female	(−)	(−)	5.0	Surgical resection	Well, 1 year
4	58	Male	(−)	(−)	6.0	Surgical resection	Died, congestive heart failure
5	44	Male	(−)	(−)	5.0	Surgical resection	Lost to follow-up
6	56	Male	(−)	(−)	10.0	Surgical resection	Lost to follow-up
7	62	Female	(−)	(−)	8.0	Surgical resection	Well, 6 years
8	37	Female	Shortness of breath, chest pain	(−)	6.0	Surgical resection	Died, pulmonary edema
9	69	Male	Shortness of breath, chest pain	(−)	7.0	Surgical resection	Died, renal insufficiency
10	59	Male	Shortness of breath, chest pain	(−)	6.0	Surgical resection	Died, congestive heart failure
11	27	Female	Unknown	(+)	5.0	Surgical resection	Died, cause unknown
12	73	Male	Shortness of breath, chest pain	(−)	10.0	Surgical resection	Died, cause unknown
13	47	Male	(−)	(−)	2.0	Surgical resection	Lost to follow-up
14	62	Female	(−)	(−)	3.1	Surgical resection	Lost to follow-up
Our case	78	Female	General fatigue	(−)	7.8	VATS, needle biopsy	Alive and well, 3 years

Our patient is the oldest to have sclerosing thymoma. All 14 cases were diagnosed by surgical resection, but we were able to make a diagnosis by video-assisted thoracic surgery, needle biopsy. *VATS* video-assisted thoracic surgery

While thymomas are the most common anterior mediastinal tumors, they are relatively rare, with an incidence of 1.5 cases per million people [12, 13]. The etiology is unknown. Approximately 30 to 50% of patients with thymoma develop comorbid myasthenia gravis [14]. Our case was not complicated with myasthenia gravis. The Masaoka staging system (modified) is most commonly used for the management of thymomas and prognosis prediction. This case was diagnosed as Masaoka stage IVa with pericardial dissemination because pericardial invasion was observed. The 5-year survival rate of patients with stage I to III thymoma is approximately 85%, and that of patients with stage IV is around 65% [15–17]. In approximately 50% of patients, thymoma is not related to the cause of death [18]. On the other hand, myasthenia gravis is related to the cause of death in approximately 20% of patients. Surgery is recommended for all surgically resectable thymoma cases (complete resection of the thymus gland and the tumor). Completeness of resection is the most important factor for prognosis. Even for stage IV thymomas, multimodality treatment with possibly complete resection is recommended [19–21]. The prognosis of sclerosing thymoma is unknown. Some cases are lost to follow-up, but some cases died of heart failure or pulmonary edema even in young patients. Since our case was diagnosed as having sclerosing thymoma (stage IVa)-like thymic amyloidoma, total thymectomy should be recommended, but she could not undergo resection due to her age and pericardial dissemination.

There are many reported cases of nephrotic syndrome as a secondary complication of malignant tumors and connective tissue disease; however, nephrotic syndrome as a complication in patients with thymoma is rare. In nephrotic syndrome associated with thymoma, many patients develop: (1) membranous nephropathy when the onset of nephrotic syndrome occurs concurrently with the thymoma; and (2) MCNS when the onset occurs after thymus resection [22]. In the present case, a definitive diagnosis of MCNS was not established since a kidney biopsy was not performed; however, based on the selectivity index, its selectivity of urinary protein was high. Therefore, MCNS was clinically diagnosed and steroid administration was started as treatment for nephrotic syndrome, which resulted in a marked reduction in tumor size. Steroid treatment has been shown to be effective in not only improving nephrotic syndrome and renal failure, but also in the regression of thymoma [23, 24]. Steroid treatment has provided successful results in thymoma, even when nephrotic syndrome is not a secondary complication. Glucocorticoid reactions that induced apoptosis of thymoma cells are thought to be triggered when steroids bind to glucocorticoid receptors in thymoma cells, resulting in the regression of thymomas [22]. There are only 14 reported cases of sclerosing thymoma in the world, and the treatment method has not yet been established.

## Conclusions

This is a case report of a patient with sclerosing thymoma-like thymic amyloidoma that showed marked reduction in tumor size when steroids were administered for paraneoplastic nephrotic syndrome. Our patient was receiving palliative treatment because resection was not possible due to her age. When hyalinized fibrous tissue is collected from a biopsy of an anterior mediastinal mass, sclerosing thymoma and amyloidoma should be considered possibilities in the differential diagnosis. Special attention must be given to tumors found in the thymus gland that are composed primarily of fibrous tissue by, for example, preparing serial sections of the entire tumor to ensure that minute thymomas are not overlooked. All reported cases of sclerosing thymomas underwent surgical resection, but steroid therapy to sclerosing thymoma has not been reported. It is still unknown whether steroid therapy is effective or not. The reports of these 14 cases of sclerosing thymomas did not indicate whether the hyalinized components were stained with Congo red. The hyalinized components of sclerosing thymoma possibly contain amyloid deposits. The marked reduction in tumor size with steroid therapy may result in amyloid deposits. The association between sclerosing thymoma and thymic amyloidoma remains uncertain. Sclerosing thymoma should be stained with Congo red. Further investigations are needed.

## Abbreviations

AA amyloidosis: Amyloid A amyloidosis
AL amyloidosis: Light chain amyloidosis
Cre: creatinine
CT: Computed tomography
DNA: deoxyribonucleic acid
IgA: immunoglobulin A
IgD: immunoglobulin D
IgG: immunoglobulin G
IgG4: immunoglobulin G4
IgM: immunoglobulin M
HIV: Human Immunodeficiency Virus
MCNS: Minimal change nephrotic syndrome
TdT: Terminal deoxynucleotidyl transferase
TTR: Transthyretin
